# No unified reward prediction error in local field potentials from the human nucleus accumbens: evidence from epilepsy patients

**DOI:** 10.1152/jn.00260.2015

**Published:** 2015-05-27

**Authors:** Max-Philipp Stenner, Robb B. Rutledge, Tino Zaehle, Friedhelm C. Schmitt, Klaus Kopitzki, Alexander B. Kowski, Jürgen Voges, Hans-Jochen Heinze, Raymond J. Dolan

**Affiliations:** ^1^Wellcome Trust Centre for Neuroimaging, University College London, London, United Kingdom;; ^2^Department of Neurology, Otto-von-Guericke University, Magdeburg, Germany;; ^3^Max Planck University College London Centre for Computational Psychiatry and Ageing Research, London, United Kingdom;; ^4^Department of Behavioral Neurology, Leibniz Institute for Neurobiology, Magdeburg, Germany;; ^5^Epilepsy-Center Berlin-Brandenburg, Department of Neurology, Charité Universitätsmedizin, Berlin, Germany; and; ^6^Department of Stereotactic Neurosurgery, Otto-von-Guericke University, Magdeburg, Germany

**Keywords:** local field potentials, deep brain stimulation, nucleus accumbens, reward prediction error

## Abstract

Functional magnetic resonance imaging (fMRI), cyclic voltammetry, and single-unit electrophysiology studies suggest that signals measured in the nucleus accumbens (Nacc) during value-based decision making represent reward prediction errors (RPEs), the difference between actual and predicted rewards. Here, we studied the precise temporal and spectral pattern of reward-related signals in the human Nacc. We recorded local field potentials (LFPs) from the Nacc of six epilepsy patients during an economic decision-making task. On each trial, patients decided whether to accept or reject a gamble with equal probabilities of a monetary gain or loss. The behavior of four patients was consistent with choices being guided by value expectations. Expected value signals before outcome onset were observed in three of those patients, at varying latencies and with nonoverlapping spectral patterns. Signals after outcome onset were correlated with RPE regressors in all subjects. However, further analysis revealed that these signals were better explained as outcome valence rather than RPE signals, with gamble gains and losses differing in the power of beta oscillations and in evoked response amplitudes. Taken together, our results do not support the idea that postsynaptic potentials in the Nacc represent a RPE that unifies outcome magnitude and prior value expectation. We discuss the generalizability of our findings to healthy individuals and the relation of our results to measurements of RPE signals obtained from the Nacc with other methods.

reinforcement learning is thought to rely on reward prediction errors (RPEs), the difference between experienced and expected rewards. RPEs are represented by midbrain dopamine neurons (e.g., Schultz et al. 1997; Cohen et al. 2012). In humans, neuronal firing rate and blood-oxygen-level-dependent (BOLD) activity in the dopaminergic midbrain and in one of its main target regions, the ventral striatum/nucleus accumbens (Nacc), both consistently correlate with RPE model predictions (Abler et al. 2006; Pessiglione et al. 2006; D'Ardenne et al. 2008; Caplin et al. 2010; Zaghloul et al. 2010). Phasic dopamine release in the rat Nacc, measured by fast-scan cyclic voltammetry, also satisfies criteria for representing RPEs (Hart et al. 2014). Value estimates used for decision making can be updated by these RPE signals. After learning, dopamine signals in response to reward-related cues often relate to the expected value associated with these cues (Fiorillo et al. 2003; Hart et al. 2015) and BOLD responses in the striatum during decision-making tasks correlate with option expected values (e.g., Tom et al. 2007).

However, latencies and durations of reward-related signals measured by human functional magnetic resonance imaging (fMRI) or by voltammetry are on the order of seconds (e.g., Hart et al. 2014; Pessiglione et al. 2006). This contrasts with the rapid and transient modulation of neuronal firing rate in dopaminergic midbrain neurons, which occurs within a few hundred milliseconds (Schultz et al. 1997). These timing differences may reflect the low temporal resolution of fMRI and voltammetry signals (Kwong et al. 1992; Venton et al. 2003). Because of this low temporal resolution, fMRI likely measures a temporally integrated, compound signal that could, in principle, correspond to a variety of distinct, although similarly energy-demanding, changes in neuronal signaling over an extended period of time. For example, measuring the BOLD signal alone permits only limited conclusions regarding the spectral composition of underlying neuronal processes (e.g., Magri et al. 2012). As a result, the two RPE components, reward and expectation, may modulate neuronal processing in the Nacc at different latencies or in distinct frequency bands even though BOLD activity and dopamine concentrations suggest that both are unified in a single RPE signal in the Nacc. Furthermore, the effects of extracellular dopamine on postsynaptic striatal neurons likely depend on the sum of concurrent striatal inputs. Taken together, fMRI and voltammetry provide an incomplete description of reward and expectation processing by Nacc neurons.

In contrast, local field potentials (LFPs) capture effective postsynaptic changes in Nacc neurons at short time scales and with high spatial specificity. The spectral profile of LFPs provides an additional data dimension along which neuronal processing of reward and expectation may differ, with potential functional implications. A particular spectral pattern in the Nacc may reflect, for example, input from other brain regions (Berke 2009; Gruber et al. 2009; Van der Meer et al. 2010), an influence of neuromodulators (Berke 2009), or a recruitment of subsets of local inhibitory interneurons or distinct striatal compartments (Berke 2009; Meer Van Deer and Redish 2009; Kalenscher et al. 2010). More generally, there is growing evidence that different frequency bands of cortical LFPs may correspond to distinct neural processing pathways (e.g., Bastos et al. 2012; Belitski et al. 2008). In principle, LFPs may thus provide direct, unique information regarding reward and expectation processing in the Nacc that is not obtained by other techniques.

LFPs from the rodent Nacc have recently received increased attention (e.g., van der Meer et al. 2010). Human electrophysiological data from the Nacc, on other hand, are rare. Here, we recorded LFPs from the Nacc during an economic decision-making task in six epilepsy patients undergoing surgery for deep brain stimulation (DBS). RPEs in similar tasks are known to correlate with the BOLD signal in bilateral Nacc (Rutledge et al. 2014). Following a standard approach in the literature (Behrens et al. 2008), we tested for two components of RPEs separately, namely for responses related to expected and experienced reward. To confirm that patients' behavior was consistent with forming a reward expectation that influenced their choices, behavior in the task was fitted by standard parametric decision models.

Given evidence of RPE signals in the Nacc from human fMRI studies and strong correlations between the BOLD signal and LFPs (e.g., Logothetis et al. 2001; Logothetis 2003; Magri et al. 2012), we expected to find signals in Nacc LFPs that are compatible with representing RPEs. Specifically, these signals were expected to occur following outcome onset and to vary in opposite directions with both gamble expected value and reward magnitude (Behrens et al. 2008; Rutledge et al. 2010). While a previous study concluded that LFPs from the human Nacc do not encode RPEs (Cohen et al. 2009a), that study employed a set of behavioral options restricted to gambles with only one fixed expected value, which limits strong conclusions. Furthermore, analyses in that study were restricted to evoked responses in the time domain. In contrast, our task included a broad range of expected values and reward magnitudes appropriate to detect RPE signals (Rutledge et al. 2014). In addition, we examined both evoked and time-frequency resolved LFPs.

## METHODS

### Patients

We recruited six patients with pharmacoresistant partial epilepsy (means ± SD age: 41.7 ± 6.6 yr; 2 females; all right handed; see Table 1 for details). One patient (P6) was excluded from all LFP analyses due to a low signal-to-noise ratio of LFPs from the Nacc in both hemispheres (see *Recording and Analysis of LFPs*). All patients participated in in-house protocols to study safety and potential anti-ictal efficacy of Nacc DBS. Clinical outcomes are reported elsewhere (Schmitt et al. 2014; Kowski et al. 2015). Both this clinical trial and the experiment reported here were approved by the Institutional Review Board of the University of Magdeburg (Registration No. 03/08). All patients gave written informed consent.

**Table 1.** Clinical data

ID	Gender/Age, yr/Disease Duration, yr	Epilepsy Syndrome	Etiology	Seizure Lateralization	Seizure Onset	AED
P1	M/39/9	Multifocal	Cryptogenic	Bilateral	Bifrontal	LCM: 400 mg ZNS: 400 mg
P2	M/32/31	Multifocal	Genetic (SCNA1)	Left, possibly bilateral	Bifrontal and left medio-temporal	OXC: 900 mg Clobazam: 5 mg STP: 4,500 mg
P3	F/52/17	Focal	Cryptogenic	Left	Temporal	LTG: 250 mg LCM: 400 mg
P4	F/44/14	Multifocal	Right hippocampal sclerosis*	Bilateral	Temporal	CBZ: 1,200 mg
P5	M/40/31	Focal	Left hippocampal sclerosis†	Left	Temporal	LTG: 400 mg LCM: 400 mg
P6	M/43/38	Left temporal	Posttraumatic lesion	Left	Temporal	LCM: 400 mg LEV: 3,000 mg

AED, antiepileptic drug; P, patient; M, male; F, female; LCM, lacosamide; ZNS, zonisamide; OXC, oxcarbazepine; STP, stiripentol; CBZ, carbamazepine; LTG, lamotrigine.

* Patient underwent right temporal lobe resection 3 yr before deep brain stimulation (DBS) surgery.

† Patient underwent left temporal lobe resection 9 yr before DBS surgery.

### Electrode Implantation

**Fig. 1.**
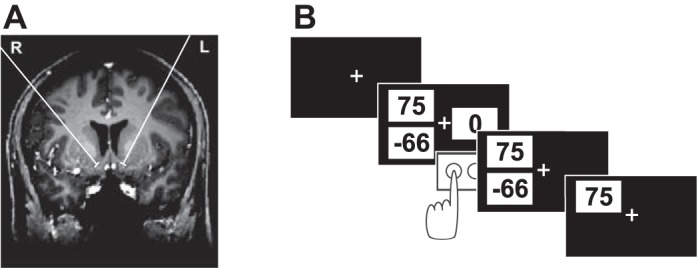
Location of deep brain stimulation (DBS) electrodes and task. *A*: exemplary presurgical MRI showing the projection of the planned placement of the 2 nucleus accumbens (Nacc) electrodes onto this MRI slice. *B*: schematic of 1 trial in which the gamble option is chosen.

All patients underwent stereotactically guided implantation of quadripolar electrodes in the bilateral Nacc and anterior thalamus. Planning of electrode placement and surgical procedures were previously described in detail (Voges et al. 2002; Zaehle et al. 2013). For the Nacc, standardized coordinates were 2 mm rostral to the anterior border of the anterior commissure at the level of the mid-sagittal plane, 3–4 mm ventral and 6–8 mm lateral of the midline. These standardized coordinates were adjusted to each individual's presurgical MRI, with the vertical limb of Broca's diagonal band as an important landmark. Electrodes were placed 2–2.5 mm lateral of the vertical limb of Broca's band, in the caudo-medial part of the Nacc. This area is thought to correspond to the remnant of the shell area in primates (Sturm et al. 2003). Intraoperative stereotactic X-ray and postoperative computer tomography were used to confirm the position of each electrode. At least two contacts of each quadripolar electrode were placed inside the Nacc, covering those parts equivalent to the Nacc shell and core regions in rodents. Figure 1*A* shows an exemplary presurgical MRI with a projection of the planned placement of electrodes.

Electrode leads were externalized for 6 days following surgery, allowing clinical test stimulation with different parameters as well as recordings from depth electrodes in various psychological tasks. Electrode cables were then connected to an impulse generator beneath the left pectoral muscle.

### Procedure and Task

Data were collected between the 3rd and 5th day postsurgery. During recording, patients completed an economic decision-making task presented on a laptop computer. We chose a task in which expected values and RPEs are known to correlate with the BOLD signal in bilateral Nacc (Rutledge et al. 2014). During the task, patients repeatedly decided whether to accept or reject a monetary gamble offer. If accepted, a gamble resulted in a monetary gain or loss with equal probabilities after a brief delay. Each patient made 200 choices between this risky gamble option and a safe option worth 0 euros. There were five different win amounts for the risky option (25, 40, 55, 75, and 100 euro cents). Loss amounts for the risky option (5–200 euro cents) were determined by multiplying gain amounts by 20 different multipliers, ranging from 0.5 to 5 to accommodate a wide range of gain-loss sensitivity. Each set of options was presented twice. Forty trials featured gamble offers with a negative mathematical expected value (calculated as the mean of the two possible outcomes of a gamble), and so subjects would lose money on average if they chose those options. The gamble expected value was positive in 150 trials. In the remaining 10 trials, the gamble offer had an expected value of zero with equal magnitudes for the potential gain and loss. The risky gamble option and the safe option were represented by three numbers on the screen, each presented in black font in the center of a white rectangle (Fig. 1*B*; the screen background color was black). Two of the three numbers were presented on one side of the screen (left or right) and corresponded to the possible gain and loss of the current gamble offer. The potential loss amount, indicated by a negative sign, and the possible win amount were presented slightly below and above the horizontal meridian of the screen, respectively, at equal eccentricities from the center of the screen. The third number, representing the safe option, was always zero and was presented on the opposite side of the screen, on the horizontal meridian. The side of the screen (left/right) on which each of the two options (safe option and gamble) was presented was counterbalanced across trials.

**Fig. 2.**
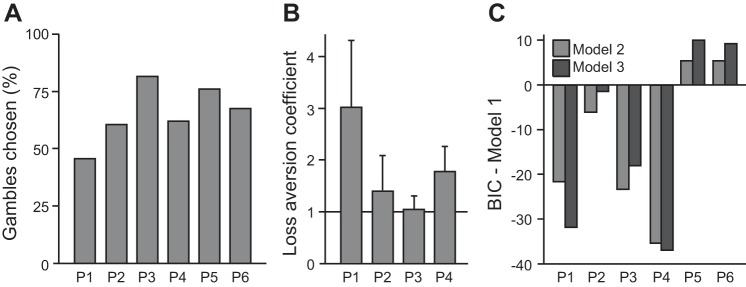
Model-based analysis of patient decisions. *A*: the 6 patients varied in their tendency to gamble, choosing a risky gamble option over the safe option in 46–82% of trials. *B*: 4 patients made decisions consistent with the value function in parametric decision models. A loss aversion coefficient >1 indicates behavioral loss aversion, a greater impact of potential losses on choice than equivalent potential gains. *C*: compared with *model 1* (which accounted for a gambling tendency but no value dependency in choice), models that included the gamble expected value (*model 2*) or a gamble subjective value that depended on a loss aversion coefficient (*model 3*) were preferred by Bayesian model comparison in 4 subjects (P1, P2, P3, and P4), which penalizes for model complexity. BIC, Bayesian Information Criterion.

Each trial started with the presentation of the two options on the screen. Patients chose the option presented on the left or on the right by pressing one of two keys on a keyboard with their left or right hand, respectively (left “Ctrl” and “Enter” on the number block, respectively). There was no time limit for decisions. If patients chose to gamble, the outcome was randomly determined by the computer and displayed for 1.5 s after a 2-s delay period. The intertrial interval was jittered between 1.5 and 2 s. The outcome of each trial counted for real money. Subjects were endowed with 15 euros at the start of the experiment and, in addition to this endowment, earned an average of 15.18 euros (range, 3.73–27.38 euros), paid out at the end of the experiment. Before the main experiment, patients practiced the task and became familiar with the range of possible gains and losses for 50 trials. Practice trials did not count for real money. Behavioral results are shown in Fig. 2.

### Recording and Analysis of LFPs

LFPs were recorded continuously from four platinum-iridium contacts (1.5 mm wide, 1.5 mm apart) on each of the four DBS-electrodes (1 in each Nacc and anterior thalamic nucleus). We report data from the bilateral Nacc, for which the task was primarily designed. Data were digitized at a sampling rate of 512 Hz using a Walter Graphtek system (data for patient P6 was recorded using Brain Vision software at a sampling rate of 2,500 Hz). Following previous work (Litvak et al. 2012), we analyzed data in a bipolar montage, in which each contact is referenced to its dorsal neighbor. This results in three channels per hemisphere. This bipolar montage maximizes spatial specificity for the Nacc by minimizing volume conduction effects from distant sources. Simultaneous to LFP recordings, data from three to four surface electrodes (Oz, Cz, Fpz and, in P2, also POz, positioned according to the 10–20 system) were obtained for patients P1 to P5.

LFPs were analyzed using FieldTrip (Oostenveld et al. 2011) and Matlab (version R2012a; Mathworks). Data were epoched from 1.5 s before options onset to 5 s after the choice (i.e., 3 s after outcome onset in trials in which patients accepted the gamble). Line noise was removed using a narrow-band, fourth-order, two-pass Butterworth filter (48.5 to 51.5 Hz and harmonics up to 250 Hz). Trials were rejected if the maximum amplitude variance across all available channels exceeded a threshold, as implemented by standard options in FieldTrip. This resulted, on average, in a rejection of 19% of all trials. Artifact-free data were baseline corrected by subtracting the mean LFP amplitude across the 100 ms that preceded the beginning of each trial (i.e., the onset of the options) from the entire epoch. The numbers of trials available for analysis were 132 (P1), 165 (P2), 186 (P3), 143 (P4), 178 (P5), and 168 (P6).

Inspection of artifact-free data of each patient revealed a low signal-to-noise ratio of Nacc recordings from patient P6. Following previous work (Cohen et al. 2009a,b), we expected an evoked response to the presentation of the two options on the screen and to the outcome of a gamble, each within the first 800 ms. While patients P1 to P5 indeed showed at least one significant positive or negative peak in both of these time windows (*P* < 0.05), trial-averaged data in none of the six Nacc channels from patient P6 differed significantly from zero in response to either event (*P* > 0.1; corrected for multiple comparisons across all time bins between 0 and 800 ms, separately for both time windows; cluster-based permutation test, see below). P6 was therefore excluded from all LFP analyses.

Spectral analysis was performed separately for low frequencies (2.5 to 40 Hz, in steps of 2.5 Hz) and high frequencies (30 to 250 Hz, in steps of 5 Hz) to account for different smoothing properties (for a similar procedure see, e.g., Buchholz et al. 2011). For low frequencies, a Fourier-transformed Hanning taper was multiplied with Fourier-transformed data segments sampled every 25 ms (window length of 400 ms). For high frequencies, discrete prolate spheroidal multitapers were used (window length of 200 ms, spectral smoothing of 20 Hz; 7 orthogonal Slepian tapers). For the analysis of phase-locked (“evoked”) responses in the time domain, the LFP signal was band-pass filtered between 0.5 and 25 Hz, following previous work (Zaehle et al. 2013) (4th-order, 2-pass Butterworth filter). Since we had no prior hypothesis regarding any difference between more ventral vs. more dorsal channels, the mean amplitude of phase-locked responses and the mean spectral power across all three channels of each electrode were used for statistical analyses. To ensure that averaging across channels did not mask any statistically significant effects in individual channels, we conducted additional analyses of all channels separately as indicated in the results section.

**Fig. 3.**
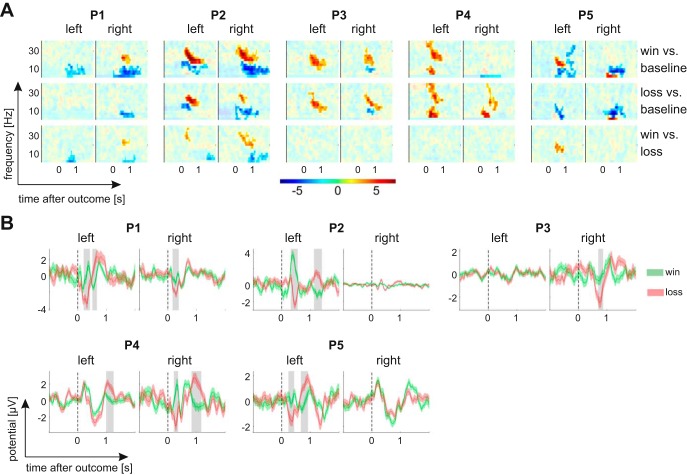
Outcome valence effects on time-frequency responses and evoked potentials. *A*: outcome valence effects on time-frequency responses up to 40 Hz (see methods for details). Color codes for *t*-values (gamble gains vs. gamble losses). Opaque areas are *P* ≥ 0.05. There is a consistent, significant increase in the power of beta oscillations within the first second after outcome onset, which is greater for gains than losses in three subjects. *B*: gain-evoked responses (green) have a greater (more positive) amplitude than loss-evoked responses (red) within the first few hundred milliseconds after outcome onset. In 4 of the 5 patients, there is also a later reversal of this effect. Shaded areas indicate significant (*P* < 0.05) time windows.

**Fig. 4.**
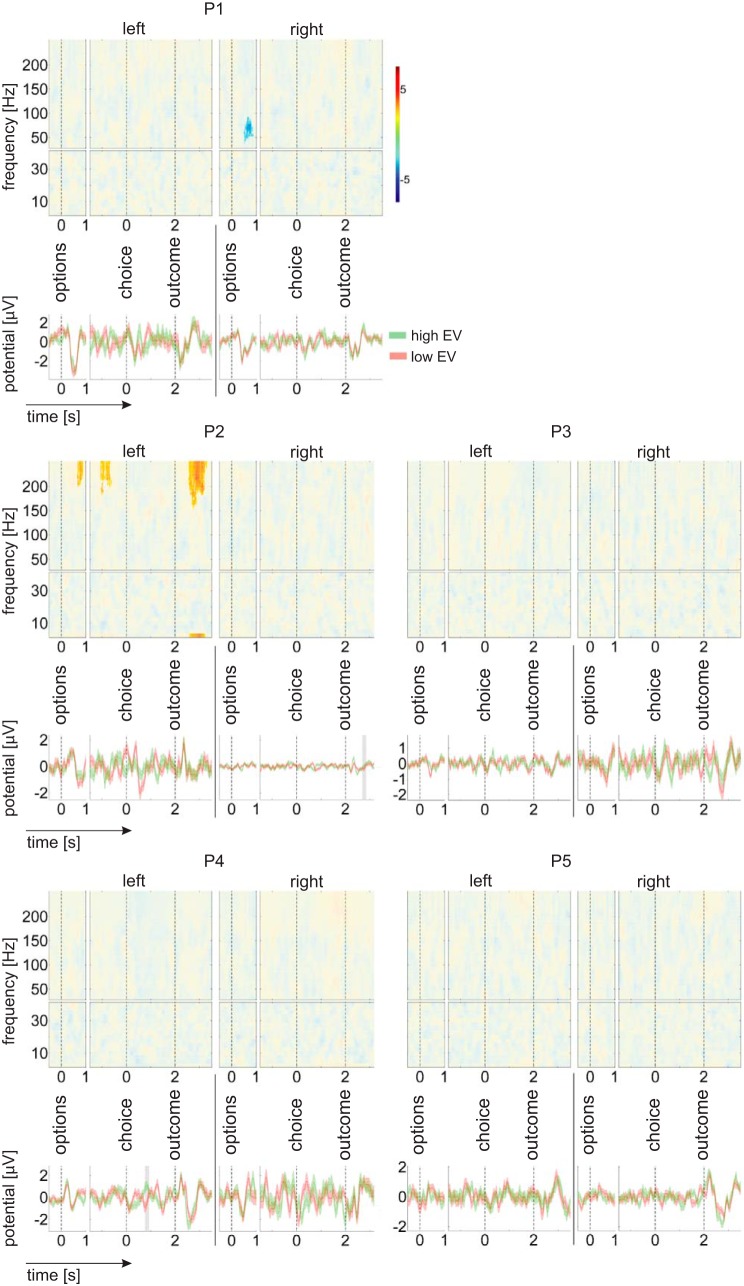
Expected value effects on time-frequency responses and evoked potentials. In the time-frequency plots, color codes for a regression *t*-statistic. Opaque areas are *P* ≥ 0.05. In the time-domain plots, the red and green time courses represent averages across trials with a relatively low or high expected value of the gamble option, respectively (as determined by a median split). Shading in the time-domain plots indicates significant (*P* < 0.05) time windows. In both time-frequency and time-domain plots, the perioption time window (at *left*, for each patient and hemisphere) includes all trials (irrespective of whether the gamble option or the safe option was chosen). The periresponse and perioutcome plots (at *right*, for each patient and hemisphere) includes only those trials in which a gamble option was chosen. Three patients show significant preoutcome expected value signals, but these signals vary in frequency and latency. Only a single patient (P2) shows an expected value signal after outcome onset, and that signal is not consistent with an expected value component of a unified RPE signal (see results). Note that raw LFP data were band-stop filtered to remove line noise (48.5 to 51.5 Hz and harmonics up to 250 Hz).

Because of the relatively small sample size in our study, group-level inference is inappropriate. However, our sample offers an excellent opportunity to study the consistency of task-induced modulations across five individual cases. We therefore report commonalities and differences between individual case results. We used a nonparametric permutation test implemented in FieldTrip to correct for multiple comparisons across time and frequency bins and both hemispheres. First, clusters comprising adjacent time bins and frequency bins (for time-frequency data) or adjacent time bins (for data in the time domain) were defined based on a threshold of the *t*-statistic for a given contrast. This *t*-statistic was computed for each time and frequency bin in each hemisphere separately and thresholded at *P* < 0.05 [independent-samples *t*-statistic (Fig. 3) or linear regression *t*-statistic (Fig. 4)]. A cluster-level statistic was obtained for each cluster by summing *t*-values across its elements (separately for clusters with positive and negative *t*-values). The null hypothesis was rejected if this cluster-level statistic exceeded a critical value, which was estimated from at least 1,000 random data partitions. To this end, trials were first randomly reassigned to the two conditions of a given contrast or, when a linear regression was used (Fig. 4), to the values of the predictor variable. The cluster-level statistic of each cluster in the actual data was then compared with the distribution of the maximum cluster-level statistic across all random data partitions under the null hypothesis of no difference between conditions (Fig. 3) or no dependency on the predictor variable (Fig. 4). We defined *P* values as the proportion of random partitions whose maximum cluster-level statistic exceeded the cluster-level statistic of each cluster in the actual data. Time- and frequency-windows for each analysis are reported together with its results. For analyses in which data from each channel were considered separately (instead of pooling across channels) clusters could span adjacent channels on the same electrode.

### Behavioral Data Analyses

Because the safe option is always worth zero, accepting gamble offers with positive expected values (average return) and rejecting offers with a negative expected value is a strategy that maximizes expected returns across trials in our task. To test whether patients gambled significantly more often when the expected value was positive vs. negative, a nonparametric resampling test was used. To this end, choices (safe or risky choice) were randomly shuffled across trials in 1,000 unique resampling iterations. For each resample, we computed the difference in the proportion of gamble choices with a positive vs. negative expected value and compared that difference to the actual observed difference for that patient. A *P* value was obtained for each individual as the fraction of resamples with a bigger difference than the observed difference.

Choice behavior was fitted with three established decision models (Kahneman and Tversky 1979; Tversky and Kahneman 1992; Tom et al. 2007; Sokol-Hessner et al. 2009; Brown et al. 2014). We estimated parameters for each patient and model using the method of maximum likelihood. *Model 1* features only a single parameter *g* that captures an overall bias to gamble independent of the values of the two options:

$$P_{\text{gamble}}=\frac{1}{1+e^{-g}}$$

In *model 2*, we tested whether choice behavior was explained by a value function where the utility of the gamble was equal to its expected value:

$$U_{\text{gamble}}=0.5{\text{V}}_{\text{gain}}+0.5{\text{V}}_{\text{loss}}$$

In this model, the probability of gambling depends on both the gamble utility and the overall tendency to gamble:

$$P_{\text{gamble}}=\frac{1}{1+e^{-\mu \left(U_{\text{gamble}}+g\right)}}$$

The parameter μ captures the sensitivity of choice probability to the utility of the gamble (the utility of the safe option is always assumed to be zero). We used Bayesian model comparison to determine whether *model 2* described choice behavior better than *model 1* after accounting for model complexity. To this end, the Bayesian Information Criterion (BIC) was computed for each patient and model. The model with the lower BIC is preferred.

Humans typically exhibit loss aversion in economic decision-making tasks: potential losses have a greater impact on choice than equivalent gains (Kahneman and Tversky 1979; Tom et al. 2007; Sokol-Hessner et al. 2009). Our experimental design also allowed us to estimate the impact on choice of losses relative to gains for each subject. To estimate the degree of loss aversion in each subject, *model 3* additionally includes a parameter λ:

$$U_{\text{gamble}}=0.5{\text{V}}_{\text{gain}}+0.5\lambda {\text{V}}_{\text{loss}}$$

A gain-loss neutral subject would have λ = 1, while a loss-averse subject would have λ > 1, and a gain-seeking subject would have λ < 1 (Sokol-Hessner et al. 2009). To determine the probability of gambling, the same equation was used as for *model 2*.

## RESULTS

Our primary goal was to test whether LFPs from the human Nacc contain signals that are compatible with representing expected value and RPE signals. To this end, we studied both phase-locked signals in the time domain and time-frequency resolved power relative to expected value, outcome valence, and outcome magnitude in an economic decision-making task in which expected value and RPE regressors are known to correlate with the BOLD signal in bilateral Nacc (Rutledge et al. 2014). To confirm that the patients in our study made choices consistent with forming value expectations, we fitted established decision models to each patient's choice behavior.

### Behavioral Modeling

In each trial, patients chose between a safe option worth zero and a risky option, a gamble in which they could win or lose varying amounts of money. On average, patients chose between the two options within 2.17 s (range of median response times, 1.46–3.04 s). Each set of options was presented twice and all six patients were more consistent than a random chooser, choosing the same way both times in >50% of option sets (range, 57–77%), with an average of 65% consistency. On average, patients chose to gamble in 66 ± 13% of trials (means ± SD; range, 46–82%; Fig. 2*A*). Because the safe option in this task is always worth zero, overall earnings are expected to be highest when gamble offers with a positive mathematical expected value (average return) are accepted and gamble offers with a negative expected value are rejected. On average, patients chose gambles with a positive expected value in 73 ± 12% (means ± SD) of trials, more often than gambles with a negative expected value, which were chosen in 42 ± 28% of trials [*t*(5) = 2.73, *P* < 0.05]. We used a nonparametric resampling test to confirm, for each patient, whether choices were influenced by the expected value of the gamble offer. Four of the six patients chose the gamble offer significantly more often when its expected value was positive vs. negative (P1: 59 vs. 8%, *P* < 0.001; P2: 68 vs. 38%, *P* < 0.001; P3: 93 vs. 43%, *P* < 0.001; P4: 75 vs. 18%, *P* < 0.001). There was no significant effect of expected value on choice in the remaining two patients (P5: 77 vs. 75%, *P* = 0.46; P6: 65 vs. 73%, *P* = 0.2).

We fitted choice behavior with three established decision models (Kahneman and Tversky 1979; Tom et al. 2007; Sokol-Hessner et al. 2013; Brown et al. 2014) and estimated parameters for each subject and model separately. *Model 1* features only a single parameter that captures an overall bias to gamble, independent of the expected value of the gamble offer. The average pseudo-*r*^2^ for this model was 0.11 (range, 0.01 to 0.21). The parameter *g* was estimated to be 0.69 ± 0.58 (means ± SD). Parameter *g* was, on average, significantly greater than zero [range, −0.16 to 1.48, *t*(5) = 2.93, *P* < 0.05], consistent with an overall tendency to gamble irrespective of gamble expected value in our subjects.

*Model 2* explains choice behavior by a value function, with the gamble utility equal to its expected value. In this model, the probability of choosing the gamble offer depends both on each patient's sensitivity to gamble utility (parameter μ) and on a patient's overall tendency to gamble (parameter *g*). The average pseudo-*r*^2^ for *model 2* was 0.18 (range, 0.07 to 0.41). The BIC was lower (preferred) for *model 2* compared with *model 1* for four of the six patients (P1, P2, P3, and P4) suggesting that these patients' decisions depended at least partly on the expected value of the gamble. Choice behavior in the remaining two patients (P5 and P6) was better explained by an overall tendency to choose gambles alone (*model 1*) according to BIC (Fig. 2*C*).

Finally, our experimental design allowed us to estimate the degree of loss aversion for each of the four patients for whom choice depends on gamble value with an additional parameter λ in *model 3*. The average pseudo-*r*^2^ for *model 3* was 0.19 (range, 0.08 to 0.41). The loss aversion coefficients for patients P1, P2, P3, and P4 were 3.03, 1.42, 1.04, and 1.78, respectively (Fig. 2*B*). The loss aversion coefficient is greater than 1 for these four patients, and the additional model complexity of *model 3* is justified by BIC for patients P1 and P4. Our results are consistent with four of the six patients using a subjective value function to guide their decision-making.

### Local Field Potentials

Following a standard approach, we tested for effects of the two RPE components separately, i.e., for an expected value effect and an effect of outcome magnitude. More specifically, if phasic dopamine release changes postsynaptic processing in the Nacc in a way that is compatible with a RPE signal, postoutcome LFPs should vary both with the outcome magnitude and its expected value, and in opposite directions (Behrens et al. 2008; Rutledge et al. 2014).

#### Outcome valence and magnitude effects.

We first tested for effects of gamble outcome on the spectral pattern of LFPs over time. In principle, any relationship between RPEs and LFPs could be driven purely by a valence effect (gain vs. loss). Alternatively, LFPs could additionally scale with outcome magnitude, as would be expected for a RPE signal. To distinguish between potential valence and magnitude effects, outcome magnitude was used as a predictor in two separate linear regression analyses, one for win trials and one for loss trials. To test for valence signals, we first compared time- and frequency-resolved power across the 2 s after outcome presentation with a baseline, separately for trials in which patients won money and for trials in which they lost [Fig. 3*A*; significance threshold *P* < 0.05 after correction for multiple comparisons across all time bins between 0 and 2 s, all frequency bins (2.5 to 40 or 30 to 250 Hz; see methods) and both hemispheres]. As a baseline, we averaged power across the inter-trial interval between −1,000 and −200 ms relative to the onset of the options.

In win trials, we found a consistent, significant increase in the power of beta oscillations (around 15 to 30 Hz) within the first second after outcome onset. This increase was present in the Nacc in at least one hemisphere of each of the five patients (Fig. 3*A*, *top row*). In addition, there was a significant modulation of the power of low frequencies (below 15 Hz) with varying direction across patients (i.e., a power decrease in P1, P2, P3, and P5 and a power increase in P4). In loss trials, power changes from baseline were less consistent and weaker. Only three patients (P2, P3, and P4) showed a significant enhancement of beta power in response to gamble losses (Fig. 3*A*, *middle row*). Furthermore, beta power was significantly higher for gamble gains vs. losses in three of the five patients (P1, P2, and P5; Fig. 3*A*, *bottom row*). No outcome valence effect was observed at higher frequencies (up to 250 Hz) in any of the patients.

To test whether spectral power was modulated by outcome magnitude, each time-frequency bin up to 250 Hz and between 0 and 1.5 s after outcome onset was regressed on outcome magnitude, separately for gamble gains and losses. Outcome magnitude modulated spectral power in two patients, but only for losses, not for gains, and only at a more liberal 10% significance threshold. Patient P2 showed an increase in power for smaller relative to larger losses between 100 and 135 Hz, 775 to 1,125 ms after outcome onset (right Nacc). Patient P3 showed a power decrease for smaller compared with larger losses in the left Nacc between 15 and 25 Hz, 750 to 1,125 ms after outcome onset [*P* < 0.1, after correcting for multiple comparisons across all channels and all time and frequency bins (separately for low and high frequencies, see methods)]. Neither following gamble gains nor losses was spectral power significantly modulated by magnitude in any of the other three patients, independent of whether the three channels of each hemisphere were analyzed separately or pooled (*P* > 0.1).

Outcome-evoked responses [event-related potentials (ERPs)] showed a similar pattern as the time-frequency responses reported above. In all five patients, the amplitude of outcome-evoked responses in the Nacc of at least one hemisphere was significantly higher following gamble gains than losses (Fig. 3*B*, *P* < 0.05 after correcting for multiple comparisons across all time bins between 0 and 2 s and both hemispheres). In addition, four patients showed a significant reversal of this valence effect on ERP amplitude at a later point (P1, P2, P4, and P5). In contrast, a modulation of ERP amplitude by outcome magnitude in the first 1.5 s following outcome onset was only found in a single patient (P1, 700 to 930 ms after the outcome), but only for gains, not for losses, and only at a more liberal 10% significance threshold. There was no ERP effect of outcome magnitude in any of the other four patients, for either gamble gains or losses (*P* > 0.1 after correcting for multiple comparisons across both hemispheres and all time bins). In summary, while we found strong and consistent outcome valence effects in all five patients (*P* < 0.05), effects of magnitude were less consistent and, if present, only detectable at a more liberal 10% significance threshold.

#### Expected value effects.

To test for expected value signals in Nacc LFP, expected value was used as a predictor in a linear regression of time- and frequency-resolved power and of the amplitude of phase-locked responses in four different time windows. These time windows were the first second after the onset of the two options, the 1.5 s before patients made a choice, the 2 s between choosing the gamble and the gamble outcome, and the first 1.5 s following outcome onset (note that the first two of these time windows may overlap in individual trials, depending on response time). If Nacc neurons respond to phasic dopamine release in a way compatible with RPEs, we should observe expected value signals following outcome onset (Behrens et al. 2008; Rutledge et al. 2014).

We found no consistent effect of expected value on LFPs following outcome onset, as would be expected for a RPE signal (Fig. 4). A modulation of postoutcome power by expected value was only observed in a single patient (P2), who showed an increase in spectral power at frequencies ≥155 Hz (550 to 1300 ms) and at 2.5 Hz (600 to 1,225 ms) with higher expected value [left Nacc; *P* < 0.05, after correcting for multiple comparisons across all time bins between 0 and 1.5 s after outcome onset, all frequency bins (2.5 to 40 Hz or 30 to 250 Hz) and both hemispheres]. This patient also showed an increase in outcome-evoked potentials with higher expected value in the right Nacc between 685 and 850 ms after outcome onset. None of the other four patients showed an expected value effect on spectral power or on the amplitude of evoked responses following outcome onset, independent of whether the three channels of each DBS electrode were pooled or analyzed separately (all *P* > 0.1).

Expected value effects on spectral power or phase-locked responses before outcome onset were observed in three of the five patients (P1, P2, and P4). However, latencies and spectral patterns of these effects varied across patients (Table 2 and Fig. 4). In the other two patients (P3 and P5), we found no effect of expected value on spectral power up to 250 Hz or on phase-locked responses in any of the time windows, independent of whether the three channels of each DBS electrode were analyzed separately or pooled (all *P* > 0.1).

**Table 2.** Effects of expected value before gamble outcome on the amplitude of phase-locked responses and time-frequency power

	Effects of Expected Value Before Outcome on...						
	Phase-locked amplitude (“evoked response”)			Time-frequency resolved power			
Patient	Side	Latency	Sign*	Side	Latency	Frequency	Sign*
P1	Left	450–610 ms postchoice†	+	Right	500–875 ms postoptions	40–90 Hz	−
P2	Left	850–1,000 ms prechoice‡	−	Left	625–1,050 ms prechoice	≥185 Hz	+
				Left	700–1,000 ms prechoice§	10–15 Hz	−
P3	Ø				Ø		
P4	Left	765–930 ms postchoice	+	Left	250 to 1,025 postchoice§	2.5–12.5 Hz	–
P5	Ø				Ø		

All *P* < 0.05 unless marked (§) after correction for multiple comparisons across both hemispheres and all time bins within each time window of interest (see *Expected value effect*s in results). Time-frequency results are also corrected across all frequency bins of interest (2.5 to 40 or 30 to 250 Hz). Note that raw local field potential data were band-stop filtered to remove line noise (48.5 to 51.5 Hz and harmonics ≤250 Hz). Ø, no significant or near-significant clusters, independent of whether all 3 channels in each hemisphere were pooled or analyzed separately (all *P* > 0.1).

* , +, and − refer to an increase and decrease in amplitude or power with expected value, respectively.

† Only when all 3 channels of each hemisphere are analyzed separately (spatial clustering across channels; not shown in Fig. 4).

‡ Only when all trials are taken into account, irrespective of choice (not shown in Fig. 4).

§ *P* < 0.1 (not shown in Fig. 4).

#### RPE effects.

To demonstrate that a signal can represent RPEs, it is not sufficient to find a significant difference between win and loss outcomes. A unified RPE signal should show a positive correlation with reward magnitude and a negative correlation with expected value or a significant pattern in the opposite direction. To test whether outcome-related signals represent a unified RPE signal, we performed a regression with a predicted RPE regressor (reward magnitude minus gamble expected value) and used a liberal *P* > 0.1 threshold both for defining clusters across time and frequency and for testing against a nonparametric distribution (see methods for details of the cluster-based permutation testing). This procedure allowed us to identify candidate signals for further examination.

We first applied this procedure to identify candidate RPE signals in LFP over time. We then tested whether these signals are better explained by the combined influence of rewards and expectations, as required of a RPE signal, or alternatively simply as an outcome valence signal that distinguishes gamble gains from losses. For each candidate RPE signal, we performed a regression that included regressors for *1*) outcome valence, *2*) reward magnitude, and *3*) gamble expected value.

For time- and frequency-resolved power up to 40 Hz, we identified candidate RPE signals with positive correlations for two subjects (P2 and P5) and negative correlations for three subjects (P1, P2, and P4). These signals overlapped with the outcome valence signals reported above and in Fig. 3*A*. Specifically, positive correlations were found with the power of beta oscillations (ranging from 12.5 to 35 Hz) between 200 and 1,175 ms after outcome onset. Negative correlations were observed for lower (2.5 to 12.5 Hz) oscillatory power between 0 and 1,125 ms after outcome onset. For the positive correlation candidate RPE signals, there was a trend for a positive correlation with reward magnitude in P5 (*P* = 0.068) but not P2 (*P* = 0.30) and no correlation with expected value in either subject (both *P* > 0.1). For the negative correlation candidate RPE signals, there were no significant correlations with either reward magnitude or expected value (all *P* > 0.1).

For time- and frequency-resolved power between 30 and 250 Hz, we observed a significant modulation by expected value and outcome magnitude only in a single patient. Unexpectedly, this patient was P5, one of the two patients whose choice behavior was not consistent with being based on value expectations (Fig. 2*C*). In this patient, power between 30 and 80 Hz, 175 to 850 ms after outcome onset, correlated positively with outcome magnitude (*P* < 0.001) and negatively with expected value (*P* = 0.009). Of the remaining four patients (P1 to P4), whose choice behavior was compatible with being based on value expectations (Fig. 2*C*), none showed evidence for a RPE signal at high frequencies that correlated either with expected value or outcome magnitude (all *P* > 0.25), let alone with both.

For outcome-evoked responses (ERP), we identified candidate RPE signals with positive correlations for all five subjects, with two potential signals at different latencies for one subject (P5). We also identified negative correlations for three subjects (P2, P4 and P5). The latencies of these outcome-evoked RPE signals overlapped with the latencies of outcome valence signals reported above and in Fig. 3*B*. Specifically, positive correlations were observed between 117 and 820 ms after outcome onset (with another cluster between 1,042 and 1,435 ms after outcome onset in P5). Negative correlations with RPE were observed between 369 and 1,357 ms after outcome onset. For the six positive correlation candidate RPE signals, there were no significant correlations with either reward magnitude or expected value (all *P* > 0.1). However, there were significant correlations with the outcome valence regressor for signals in all five subjects (*P* < 0.05; the later candidate signal in P5 was significant but not the earlier signal that had a positive relationship at *P* = 0.12). This result suggests that outcome-evoked responses, which were positively correlated with RPE regressors, are better explained as outcome valence signals than as RPE signals.

For the three negative correlation candidate RPE signals, there was also no significant correlation with either reward magnitude or expected value (all *P* > 0.1). However, there were significant negative correlations with the outcome valence regressor in all three subjects (*P* < 0.05). This result suggests that outcome-evoked responses negatively correlated with RPE regressors are better explained as outcome valence signals than as RPE signals.

## DISCUSSION

We tested whether LFPs in the human Nacc contain signals that are compatible with a RPE. RPE signals are expected to emerge after the outcome of a risky choice is revealed and to vary both with outcome value and with a prior value expectation (in opposite directions; Behrens et al. 2008). Human fMRI studies (e.g., Abler et al. 2006; Pessiglione et al. 2006; Rutledge et al. 2010), voltammetry recordings in rodents (Hart et al. 2014), and a recent study of the firing rate of human Nacc neurons (Patel et al. 2012) support the idea that RPE signals are present in the Nacc. In contrast, we found little evidence for RPE signals in LFPs recorded from the human Nacc during an economic decision-making task in which BOLD activity in bilateral Nacc should represent RPEs, based on the results of previous studies using a similar task design (Sokol-Hessner et al. 2013; Rutledge et al. 2014). Four of the patients in our study made choices in a way that required them to consistently form value expectations (Fig. 2), which were reflected in Nacc LFPs in three of these patients before outcomes were revealed (Fig. 4 and Table 2). In contrast, none of these patients showed a modulation of Nacc LFPs after outcome onset that unified expected value and outcome magnitude, as expected for a RPE signal.

### Little Evidence for a Unified RPE Signal in LFPs in the Human Nacc

Since RPEs depend on value predictions, we expected to find RPE signals in the four patients whose choice behavior strongly indicated that they formed such value predictions (P1 to P4, Fig. 2*C*). The absence of a RPE signal in all four of these patients clearly speaks against RPE signals in Nacc LFPs and contrasts with previous fMRI findings in a similar task (Rutledge et al. 2014). However, patient P5 did show a high-frequency signal compatible with representing RPEs, despite no evidence for the behavior of that patient being guided by value expectations. Since the behavior of patient P5 suggested that he was not paying attention to the potential gamble outcomes, it is difficult to speculate on the functional significance of this finding. It would be interesting for future research to test patients in a learning task in which many subjects fail to make choices consistent with using RPEs to update value estimates. In such a task, RPE signals have been measured with fMRI in the striatum of learners but not nonlearners (Schönberg et al. 2007) and accumbens potentials might similarly differ between learners and nonlearners.

Reports of RPE signals in the phasic dopaminergic input to the Nacc (Hart et al. 2014) and in Nacc output (Patel et al. 2012) might imply that the two signals are causally related, i.e., that RPEs expressed in neuronal spiking originate from postsynaptic effects of phasic dopamine at the time of the outcome. However, in principle, the neuronal firing rate in the Nacc may reflect changes in postsynaptic excitation integrated over a longer period of time, including changes that occur before an outcome is revealed. Indeed, human fMRI studies suggest that the Nacc is sensitive to a value expectation before outcome onset (Knutson et al. 2005; Rutledge et al. 2014). Accordingly, RPE signals in the Nacc output could, in principle, reflect an integration of current, outcome-related information with an anticipatory modulation of neuronal excitability by expected value before the outcome. Because fMRI has a relatively low temporal resolution (Kwong et al. 1992) and is sensitive not only to postsynaptic changes but also to multiunit spiking (albeit to a lesser degree; e.g., Logothetis 2003), it may be difficult to distinguish these two cases on the basis of the BOLD signal alone. Here, studying LFPs from the human Nacc, we found signals that varied with expected value after the outcome in only one of the four patients who made choices consistent with forming a value expectation. Expected value signals before the outcome, however, were observed in three of these patients. The heterogeneity of expected value signals across patients (Table 2) suggests a greater variability in the processing of reward and expected value than implied by fMRI. Furthermore, in the one patient who adjusted choices to expected value and who showed postoutcome signals that varied both with the actual outcome and its expected value, outcome and expectation effects occurred in nonoverlapping frequency bands and at distinct latencies (Figs. 3 and 4). Taken together, we found little evidence of a unified RPE signal in LFPs from the Nacc, in the sense of a single postoutcome signal, defined in frequency and time, that varies both with outcome magnitude and option expected value.

Neuronal signals in the Nacc are often implicitly assumed to reflect dopaminergic input from the midbrain (e.g., O'Doherty et al. 2004; Patel et al. 2012). However, in agreement with our own findings, this assumption has been questioned on empirical grounds, and a more integrative role of the Nacc has been proposed, specifically in action selection. For example, Klein-Flügge et al. (2011) tested subjects with a task that dissociated a stimulus feature that was relevant for instrumental learning (timing) from the immediately rewarding aspect of that stimulus. They found that the fMRI signal in midbrain regions, but not in the ventral striatum, was compatible with a RPE. The ventral striatal signal instead reflected the accuracy of timing predictions, i.e., the variable important for instrumental learning in their task. The idea of a ventral striatal update of behaviorally relevant variables (“policy update”), which do not necessarily have to include value, receives further support from fMRI studies that correlate striatal signals with experienced vs. foregone action outcomes (FitzGerald et al. 2014), in particular when their probabilities are varied independently (Li and Daw 2011).

A more general role for the Nacc in task-dependent policy updating, rather than a restricted function in reward prediction and error signaling, is compatible with a broader view of its contribution to action selection. According to an influential theoretical model, the Nacc integrates behaviorally relevant information from different regions, including the dopaminergic midbrain, hippocampus, amygdala, and ventromedial prefrontal cortex (Grace 2000; Goto and Grace 2008; Haber and Knutson 2010). By gating these afferent inputs, the Nacc could contribute to behavioral choice (Grace 2000; Gruber et al. 2009; Calhoon and O'Donnell 2013), particularly when choice requires knowledge and updating of instrumental contingencies under ambiguity (Floresco 2015). In our task, contingencies were made explicit before the experiment, and no learning was required. Indeed, all patients were trained on the task for 50 trials before they started the experiment. The explicit probabilities present in our task enabled us to systematically vary the potential gains and losses from trial to trial so that subjects would have to form a new value expectation on each trial to maximize earnings.

A RPE signal in the Nacc has been observed in fMRI data recorded during similar tasks that did not require learning (Rutledge et al. 2014; Sokol-Hessner et al. 2013) and in simpler tasks with explicit probabilities (Abler et al. 2006; Rutledge et al. 2010). Further studies are needed to test whether Nacc LFPs correlate with prediction errors when contextual changes necessitate behavioral updates, i.e., instrumental learning, and could also reveal whether any learning-related signals actually depend on value.

### Outcome Valence Signals in LFPs in the Human Nacc

While spectral patterns and latencies of expected value signals varied across patients (Fig. 4 and Table 2), we observed strong and consistent valence signals following outcome onset. Specifically, outcomes induced a beta enhancement within the first second after outcome onset in the Nacc in at least one hemisphere in all five patients. This replicates findings in a previous study of human Nacc LFPs (Cohen et al. 2009b). However, unlike in Cohen et al. (2009b), who used group-level inference across six patients, this beta enhancement distinguished between outcomes, i.e., it was significantly larger for gains than for losses in three patients. Furthermore, and in agreement with Cohen et al. (2009b), the amplitude of outcome-evoked responses also distinguished between gains and losses, with an additional reversal in the polarity of this difference at later latencies (Fig. 3). Taken together, outcome-induced beta oscillations and outcome-evoked potentials represent a reliable, strong pattern of outcome processing in the human Nacc.

### Limitations

#### Impact of clinical disorder and medication.

Recording LFPs from the human Nacc requires a clinical indication for DBS therapy. In our study, LFPs were recorded from patients with pharmacoresistant partial epilepsy. In all patients, video-EEG-monitoring revealed a focal (i.e., cortical) epileptogenic zone. The Nacc can be involved in seizure propagation (Deransart et al. 2000; Schmitt et al. 2014), and the clinical rationale underlying DBS of the Nacc in epilepsy is a suppression of seizure propagation, not of an epileptogenic focus. Patients were constantly under supervision during recordings, and no seizure was observed or reported in any case. No inter-ictal epileptic activity was observed in the recorded data.

We cannot exclude the possibility that RPE coding is altered in epilepsy and that this explains the discrepancy between our findings and previous fMRI studies (e.g., Rutledge et al. 2014). However, our patient population had etiologically and clinically heterogeneous epilepsy syndromes (Table 1) and this reduces the likelihood of this possibility. Furthermore, two previous studies found no abnormality in choice behavior of patients with temporal lobe epilepsy (like 3 of the patients in our study) in risky decision-making tasks that were similar to our paradigm (Labudda et al. 2009; Delazer et al. 2010). Indeed, choice behavior of four of the patients in our study was consistent with standard parametric decision models and showed levels of loss aversion that are within the range of normal behavior established in healthy participants (Sokol-Hessner et al. 2009), evidence that they form the value expectations necessary for expression of RPE signals.

All patients were taking standard anticonvulsant medication at the time of data acquisition (Table 2), with different drugs acting predominantly via a blockade of ion channels (LCM, LTG, ZNS, CBZ, and OXC) and/or via interference with GABAergic neurotransmission (STP and Clozabam). We cannot rule out any potential alteration of neuronal signals in the Nacc by these drugs. However, the heterogeneity in medication (Table 2) contrasts with the consistency of valence effects across patients (Fig. 3) and across studies of patients with distinct disorders (Cohen et al. 2009b). A selective and uniform change in the processing of value expectations across the different pharmacodynamics seems unlikely.

#### Analyzed data features.

We examined both phase-locked, time-domain data and time-frequency resolved power across wide time and frequency windows likely to capture both outcome evaluation and the processing of a value expectation. We used a nonparametric cluster-based test within each individual instead of underpowered group-level statistical inference. To avoid type II errors, we repeated analyses before and after pooling data across the three channels of each hemisphere and at a more liberal 10% significance threshold. Time-frequency data were explored up to the limit set by our sampling frequency (512 Hz). While we cannot exclude the possibility that we missed expected value signals at higher frequencies, the 250-Hz limit in our study is close to the ∼300-Hz boundary that is commonly used to separate LFPs from multiunit activity (e.g., Logothetis 2003). Finally, to remove noise artifacts, we used a narrow band-stop filter ∼50 Hz (and harmonics), which could, in theory, have masked RPE signals if those signals were confined to the narrow frequency bands affected by the filter. However, physiological effects on spectral power in the gamma band (>∼40 Hz) typically span 10 Hz or more (e.g., Bauer et al. 2014; see Cohen et al. 2009c, for an example of Nacc gamma oscillations), and peak frequencies of gamma oscillations typically vary across individuals (e.g., Muthukumaraswamy et al. 2009). Therefore, it seems unlikely that narrow band-stop filtering would significantly interfere with our ability to detect RPE signals across multiple patients.

#### Behavioral paradigm.

Like many fMRI studies (Tom et al. 2007; Sokol-Hessner et al. 2013; Rutledge et al. 2014), our paradigm does not vary the probability of reward associated with a gamble. However, some studies do vary reward probabilities (e.g., Abler et al. 2006; Rutledge et al. 2010). Our results are inconsistent with a RPE representation in Nacc LFPs along the lines of the dominant view (Schultz et al. 1997), which proposes that RPE signals should reflect the magnitude of outcomes. Future studies could test whether Nacc LFPs show patterns consistent with representing RPEs when probabilities but not magnitudes are varied, and a positive result combined with the negative result we present here would suggest that Nacc LFPs might represent a more limited form of error signal than the RPE signals that dopamine neurons are commonly believed to represent (Schultz et al. 1997; Caplin et al. 2010).

In conclusion, LFPs from the human Nacc show strong and consistent signals reflecting outcome valence, but heterogeneous, inconsistent expected value signals that emerge, at least in some subjects, before an outcome is revealed. Because of nonoverlapping latencies and spectral patterns of outcome and expected value effects in patients whose behavior reflects value expectations, our data do not support the idea of a unified RPE signal in Nacc LFPs that is driven by the phasic dopamine release known to represent RPEs. Instead, RPE coding in the spiking activity of Nacc neurons may reflect an integration of changes in neuronal processing that occur both before and after an outcome is revealed.

## GRANTS

This work was supported by the Wellcome Trust (Ray Dolan Senior Investigator Award 098362/Z/12/Z). M.-P. Stenner was supported by a scholarship from the German Research Foundation (Deutsche Forschungsgemeinschaft DFG, STE 2091/1-2). R. B. Rutledge was supported by the Max Planck Society. T. Zaehle, J. Voges, and H.-J. Heinze received funding from the German Research Foundation (Deutsche Forschungsgemeinschaft, Sonderforschungsbereich SFB-779 TPA2). The Wellcome Trust Centre for Neuroimaging is supported by core funding from the Wellcome Trust 091593/Z/10/Z.

## DISCLOSURES

No conflicts of interest, financial or otherwise, are declared by the author(s).

## AUTHOR CONTRIBUTIONS

Author contributions: M.-P.S., R.B.R., T.Z., F.C.S., H.-J.H., and R.J.D. conception and design of research; M.-P.S. and R.B.R. analyzed data; M.-P.S. and R.B.R. interpreted results of experiments; M.-P.S., R.B.R., and K.K. prepared figures; M.-P.S. and R.B.R. drafted manuscript; M.-P.S., R.B.R., F.C.S., and R.J.D. edited and revised manuscript; M.-P.S., R.B.R., T.Z., F.C.S., K.K., A.B.K., J.V., H.-J.H., and R.J.D. approved final version of manuscript; T.Z., A.B.K., and J.V. performed experiments.
